# Acute Hepatitis after Ingestion of a Preparation of Chinese Skullcap and Black Catechu for Joint Pain

**DOI:** 10.1155/2016/4356749

**Published:** 2016-04-10

**Authors:** Charilaos Papafragkakis, Mel A. Ona, Madhavi Reddy, Sury Anand

**Affiliations:** ^1^Department of Gastroenterology, Hepatology and Nutrition, MD Anderson Cancer Center, Houston, TX 77030, USA; ^2^Department of Gastroenterology & Hepatology, The Brooklyn Hospital Center, Brooklyn, NY 11201, USA

## Abstract

Many herbal preparations are routinely used and have been occasionally associated with a wide range of side effects, from mild to severe. Chinese skullcap and black catechu are herbal medications commonly used for their hepatoprotective and other properties. We report a case of acute toxic hepatitis associated with ingestion of Chinese skullcap and black catechu in one preparation for the alleviation of joint pain.

## 1. Introduction

Numerous herbs are commonly used worldwide for medicinal use. Despite their established benefits, they can potentially have deleterious effects. We report a case of acute toxic hepatitis caused by ingestion of Chinese skullcap and black catechu, which have been reported to have hepatoprotective properties among others. In our case the patient was taking a preparation of both herbs for arthralgias.

## 2. Case Report

A 54-year-old woman presented with generalized pruritus, diarrhea, weakness, and jaundice for two weeks. She described the diarrhea as nonbloody, watery, and associated with mild abdominal cramps. Her urine was dark but she denied any change in the stool color. She denied recent hospitalizations, history of travel, or contact with anyone with infectious hepatitis. Also, she reported weight loss of approximately four pounds during this period. Her past medical history included hypertension, polyarthritis with arthralgia, and osteoporosis for which she was taking bisoprolol, oral bisphosphonate, calcium, and vitamin C. She denied drinking alcohol, intravenous drug use, blood transfusions, or needle-stick injuries. She had been in a monogamous relationship and reported barrier-protected sexual contact. On admission, the patient was icteric with no other signs of chronic liver disease. Her vital signs were normal and she was afebrile. On physical examination, she had a palpable liver 3-4 centimeters below the costal margin at the midclavicular line with no clinical evidence of portal hypertension. The remainder of the physical examination was normal. Laboratory tests revealed normal blood count and basic metabolic panel. Liver tests showed aspartate aminotransferase (AST) level of 1131 IU/L [reference range: 8–34 IU/L], alanine aminotransferase (ALT) 2312 IU/L [6–55 IU/L], alkaline phosphatase (ALP) 285 U/L [40–150 IU/L], total bilirubin of 12.8 mg/dL [0.2–1.2 mg/dL], and serum creatinine of 0.4 mg/dL [0.7–1.3 mg/dL]. Amylase was 96 mg/dL [25–125 U/L] and lipase 430 U/L [8–78 U/L]. Prothrombin time was normal, INR was 1.0 [0.8–1.2], and partial thromboplastin time was 40 seconds [9.8–11.7 seconds]. Stool studies for ova and parasites were negative. Serological testing for acute infectious hepatitides A, B, and C was negative, except for hepatitis A anti-IgG which was positive indicating previous exposure. Cytomegalovirus and Epstein Barr virus tests were also negative. Serological studies for autoimmune hepatitis, anti-nuclear antibodies, anti-mitochondrial antibodies, anti-smooth muscle antibodies, and anti-liver/kidney microsomal antibodies were negative. An ultrasound of the liver showed dilated intrahepatic biliary ducts with a normal sized common bile duct. Hepatobiliary iminodiacetic acid scan and magnetic resonance cholangiopancreatography were normal, except for mildly dilated intrahepatic biliary ducts. No liver biopsy was performed in our patient.

She was empirically treated with ursodeoxycholic acid and ciprofloxacin for pruritus and diarrhea, respectively. On the third day of hospitalization, laboratory tests were improved (Table 1). Her diarrhea and pruritus resolved; however, her jaundice lagged behind. The comprehensive workup did not reveal an etiology for her hepatitis. However, upon further questioning, the patient stated that she was taking a preparation of Chinese skullcap and black catechu for her joint pain for two to four weeks prior to admission. The liver enzyme abnormalities were attributed mainly to the skullcap. Significant improvement of her symptoms and downward trend of the liver enzymes obviated the need for liver biopsy or further workup. She was advised to discontinue the skullcap and discharged home. She continued taking ursodeoxycholic acid with subsequent resolution of her symptoms and improvement in liver biochemistries.

## 3. Discussion

Chinese skullcap, also known as* Scutellaria baicalensis*, is a member of the mint family and is widely used in alternative medicine. It was anecdotally used in the eighteenth century under the name “mad-dog weed,” to treat rabies. Its root contains the flavonoids baicalin, wogonin, and baicalein. Reported to have hepatoprotective [1], antianxiety [2], anti-inflammatory [3], antihypertensive [4], and anticancer [5] properties, it has also been used to treat premenstrual syndrome, insomnia, muscle spasms, headaches, jaundice, urinary tract infections, and gout. Reports show that baicalin can reduce the absorption of cyclosporine [6] and statins [7]. There is no current clear evidence that it is safe during pregnancy, nursing women, and children. Acute thrombocytopenic purpura [8], pulmonary complications such as pneumonitis [9], and a combination of hepatotoxicity and bilateral reticulonodular pulmonary infiltrates [10] have been reported in association with skullcap ingestion.

Few case reports exist in the literature regarding the use of the preparation syo-saiko (xiao-chai-hu-tang in Chinese) [11, 12], which is a mixture of bupleurum root, pinellia tuber,* Scutellaria* root, jujube fruit, ginseng root, glycyrrhiza root, and ginger rhizome, and to our knowledge only three reports of acute toxic hepatitis related to ingestion of Chinese skullcap and black catechu have been elucidated [13, 14].

Black catechu is also known as cutch, dark catechu, or acacia catechu. It is found in the regions between the Himalayas and southern India. Derived from a small tree, it mainly consists of catechin, catechutannic acid, and taxifolin. Catechin is used for its hemostatic properties and taxifolin is believed to have hepatoprotective, antifungal, antibacterial, anti-inflammatory, antioxidant, and antidiarrheal properties. A study showed the ethyl acetate extract of acacia catechu was hepatoprotective and inhibited diarrhea and carbon tetrachloride-induced liver injury in albino rats [15]. Hypotension has been reported and is likely due to release of bradykinin and other vasodilatory chemicals.

Traditional Chinese herbs and medicines are widely used and circulate essentially unregulated outside China, where strict regulations are applied. Occasionally, they are found to be contaminated with other elements, for example, dust, pollens, insects, heavy metals, and toxins [16].

Definitive association of liver injury due to traditional Chinese medicines or herbs is an ongoing matter of debate. As reported in a recent study, 65 commonly used herbs, drugs, and supplements and 111 traditional Chinese medicines, herbs, or herbal mixtures have been associated with liver injury, however, with low level of evidence supporting this association [17].

A recent review by Teschke et al. of 77 publications on 57 different herbs and mixtures of traditional Chinese medicine demonstrated that many of them (28 out of 57) were associated with liver injury [16]. It is common practice to report a Chinese medication as hepatotoxic based on ALT and ALP levels. However, suggesting that a traditional Chinese medication is hepatotoxic based only on elevated liver chemistries has not been shown to be entirely appropriate to confirm such an association. Therefore, it has been suggested to use a liver specific causality assessment algorithm. The Roussel Uclaf Causality Assessment Method (RUCAM) is a standardized and validated quantitative grading tool that improves case data evaluation and provides higher quality classification of probable and highly probable cases of liver toxicity due to traditional Chinese medicines [18]. The RUCAM has two subscales to differentiate between hepatocellular type and cholestatic or mixed type through the use of ratio *R* (ALT/ALP). Our case meets criteria for Hy's Law for drug-induced liver injury (ALT > 3x ULN and Tbili ≥ 2x ULN, excluding other potential causes of liver injury) [19]. The updated RUCAM score was calculated to be 8, which makes the association of this herbal mixture with liver injury “probable.”

## 4. Conclusion

Traditional Chinese medicines and herbs are widely used. Daily practice has shown that herbal medications may cause medical conditions that occasionally require hospitalization and close monitoring. Patients often do not consider herbal supplements as actual medications and are unaware of their potential health hazards. It is important for clinicians to conduct thorough histories, inquire about supplement use whenever clinical suspicion is high, and use a standardized, validated assessment method, such as the RUCAM, in order to assess causality of liver toxicity due to Chinese herbs or medicines. Surveillance for adverse side effects is crucial for the patient and care provider.

## Figures and Tables

**Table 1:** Trend of liver chemistries during hospitalization.

	Day 1	Day 2	Day 3	Day 4	Day 5	Day 6	Day 7
AST (IU/L)	1131	950	811	810	682	548	496
ALT (IU/L)	2012	1893	1603	1492	1544	1229	1098
Alkaline phosphatase (IU/L)	285	298	276	293	316	311	282
Total bilirubin (mg/dL)	12.8	11.7	11.1	10.9	11.4	7.9	5.7
Direct bilirubin (mg/dL)	9.3			9.5			
Albumin (g/dL)	2.6	2.8	2.5	2.6	3.0	2.8	2.9
